# Live and Heat-Killed Probiotic *Lactobacillus paracasei* PS23 Accelerated the Improvement and Recovery of Strength and Damage Biomarkers after Exercise-Induced Muscle Damage

**DOI:** 10.3390/nu14214563

**Published:** 2022-10-30

**Authors:** Mon-Chien Lee, Chin-Shan Ho, Yi-Ju Hsu, Chi-Chang Huang

**Affiliations:** Graduate Institute of Sports Science, National Taiwan Sport University, Taoyuan City 333325, Taiwan

**Keywords:** *Lactobacillus paracasei* PS23, probiotic, heat-killed bacteria, muscle damage, exercise

## Abstract

Excessive, high-intensity or inappropriate exercise may cause muscle damage. How to speed up recovery and reduce exercise discomfort are currently very important issues for athletes and sports people. Past research has shown that probiotics can improve inflammation and oxidative stress, as well as improve exercise performance and antifatigue. However, further research is needed to confirm the recovery benefits for muscle damage. In this double-blind design study, all subjects were randomly assigned to placebo, a live *Lactobacillus paracasei* group (L-PS23, 2 × 10^10^ colony forming unit (CFU)/day), or a heat-killed *L. paracasei* group (HK-PS23, 2 × 10^10^ cells/day), and supplemented for six consecutive weeks. Afterwards, subjects completed 100 maximal vertical jumps to bring about exercise-induced muscle damage (EIMD). Countermovement jump (CMJ), isometric mid-thigh pull (IMTP), and Wingate anaerobic test (WAnT), as well as blood tests for markers of muscle damage and inflammation were made pre-exercise and 3, 24, 48 h post exercise. The results show that both L-PS23 and HK-PS23 supplementation significantly slowed the loss of muscle strength after muscle injury, and they significantly reduced the production of markers of muscle damage and inflammation (*p* < 0.05). In addition, L-PS23 and HK-PS23 had the benefits of accelerating the recovery and improvement of muscle strength, the blood markers of muscle injury and inflammation, and slowing the decline in testosterone concentrations (*p* < 0.05). Especially in the HK-PS23 supplemented group, there was a better trend. In conclusion, we found that L-PS23 or HK-PS23 supplementation for six weeks prevented strength loss after muscle damage and improved blood muscle damage and inflammatory markers, with protective, accelerated recovery and anti-fatigue benefits.

## 1. Introduction

Exercise-induced muscle damage (EIMD) occurs after strenuous or prolonged exercise, especially when eccentric muscle contractions are involved [1] and is characterized by delayed-onset muscle soreness (DOMS), structural damage, acute local inflammation and changes in biochemical parameters [2], such as elevated creatine kinase (CK), lactate dehydrogenase (LDH), and myoglobin (Mb) [3], as well as elevated inflammatory and oxidative markers [C-reactive protein (CRP), malondialdehyde (MDA), etc.] [4]. These symptoms persist for days and are accompanied by a decline in muscle strength and performance, leading to fatigue, which can negatively impact performance in athletes and amateurs [5]. For EIMD in professional athletes, mild or moderate DOMS is induced within 2 to 3 days of athletic training. However, in an untrained population, EIMD can lead to severe DOMS and loss of muscle function, in which case a longer recovery period is required to fully repair the muscle [6]. Therefore, in addition to an appropriate training program, different strategies must be undertaken to delay or reduce muscle fatigue and improve the fitness and rapid recovery benefits of training in response to high-intensity training or consecutive high-intensity competitions [7].

Strategies such as appropriate rest, physical therapy, cryotherapy, compression garments, and nutritional supplements are known to be effective in helping to reduce the effects of EIMD or speed recovery from fatigue [8]. Of these, nutrition-based interventions targeting post-exercise inflammation and oxidative stress have received considerable attention [9]. Intervention through nutritional supplements can not only accelerate high-performance athletes’ recovery from DOMS or muscle fatigue but can also benefit amateur athletes by enabling them to train more frequently or reduce injury risk, among other benefits that may exist [10]. Currently popular sports nutrition supplements primarily include nutritional supplements with antioxidant or anti-inflammatory properties, such as curcumin [11], omega-3 fatty acids [12], and polyphenolic extracts [13]. However, in recent years, as the role of the gut-muscle axis has received increasing attention, more and more studies have explored the relationship between gut microbes and exercise performance [14]. The purported anti-inflammatory effects of probiotics have received considerable attention in the sports world [15], with numerous studies showing that supplementation with probiotics can improve exercise performance, delay fatigue, and speed recovery from muscle damage [16,17].

Probiotics are defined as “live microorganisms that, when administered in sufficient amounts, confer host health benefits, and different strains have different efficacy specificities” [18]. However, recent studies have suggested that non-viable probiotics can still evoke immune responses, through cell surface components including exopolysaccharides, peptidoglycan, and lipoteichoic acid, and produce beneficial biological responses, in addition, they are associated with greater safety, standardization and ease of preparation, which is better than that of live probiotics [19]. The term “postbiotics” refers to non-viable or inactivated bacterial cells that, when administered in sufficient amounts, likewise provide benefits to consumers [20]. Previous research has shown that heat-killed bacteria can reduce inflammation by boosting the immune response [21]. A study of athletic performance found that heat-killed bacteria reduced the activity of CK and LDH in the blood and delayed post-exercise fatigue in athletes [22].

It has been reported that the gut microbiota of athletes has higher diversity and abundance of bacterial species, which is associated with exercise performance, and increased protein intake compared to sedentary individuals [16]. A growing number of studies has shown that probiotic supplements can modulate the gastrointestinal function and upper respiratory tract symptoms regulating the immune system [23]. More recent evidence suggests that the intervention of probiotics can enhance exercise performance in athletes and recreational subjects [23,24,25].

*Lactobacillus paracasei* PS23 was previously isolated from the feces of healthy humans [26]. Past studies in animals have found that *L. paracasei* PS23 improves gastrointestinal function by modulating gut microbiota (GM) and has anti-colitis properties [27,28]. Furthermore, *L. paracasei* PS23 modulated age-related cognitive decline by reducing reactive oxygen species (ROS) and inflammation levels, and by altering the effects of the gut–brain axis [29]. Both live and heat-killed PS23 (HK-PS23) (10^8^ cells/0.2 mL/day) reversed mice chronic corticosterone-induced anxiety and depression-like behaviors [30]. On the other hand, supplementation with PS23 (10^8^ or 10^9^ cells/0.2 mL/day) improved mice mitochondrial performance, IL-10, antioxidant enzymes and protein uptake, and reduced inflammatory cytokines and ROS, thereby improving the age-related decline in muscle mass and strength [31]. In addition, HK-PS23 alleviated the muscle function of aging mice via ghrelin stimulation [26]. Although PS23 has been found to improve muscle mass and strength in aging mice in past animal experiments, there are no studies that have shown that supplementation with PS23 in young adults has an effect on athletic performance or slowing muscle damage. Therefore, in the current study, we aimed to investigate the protective and restorative effects of live PS23 (L-PS23) and HK-PS23 supplementation for six consecutive weeks on physical performance, muscle damage, and inflammation indicators after EIMD.

## 2. Materials and Methods

### 2.1. Sample Preparation

*L. paracasei* PS23 (DSM 32322) was previously isolated from healthy human feces. The L-PS23 treatment capsule contained 100 mg of powdered live *L. paracasei* PS23, equivalent to 10 billion CFU of L-PS23, while the HK-PS23 treatment capsule contained 100 mg of powdered heat-killed *L. paracasei* PS23, equivalent to 10 billion cells of HK-PS23. The placebo capsule contained only the microcrystalline cellulose (MCC) and was matched to the L-PS23 and HK-PS23 treatment capsule for size, color, and taste. All subjects were asked to take two capsules per day for 6 weeks.

### 2.2. Participants

**Figure 1:**
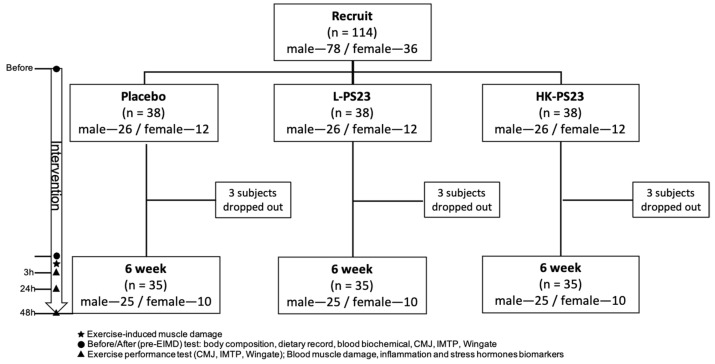
Experimental procedure description.

The study was conducted according to the guidelines of the Declaration of Helsinki. Experiments were performed after approval by the Institutional Review Board of Landseed International Hospital (Taoyuan, Taiwan; LSHIRB No. 22-003-A2) had been received. This trial was registered at clinicaltrials.gov (accessed on 6 October 2022) as NCT05346432. Males and females aged 20–40 years old were included in the study. Anyone with at least one of the following characteristics was excluded: (1) a history of cardiovascular disease, hypertension, metabolic disease, asthma, or cancer; (2) BMI ≥ 27; (3) 6 months with limb, neuromuscular motor impairment, etc., meaning the subject cannot engage in exercise; (4) having taken anti-inflammatory, analgesic, acute and chronic disease drugs or other drugs in the last month; (5) students or stakeholders of the principal investigator; (6) those who smoke or drink; (7) having used probiotic powder, capsules or lozenges (including yogurt, and other related foods) within two weeks; (8) food or lactic acid bacteria product allergy; (9) having undergone hepatobiliary gastrointestinal surgery (except hernia and polypectomy); (10) no specific sport or training activity in the past 1 year, such as basketball, volleyball, etc., involving multiple jumps or leg weight training. A total of 114 subjects from the National Taiwan Sport University were included in the trial, including 78 males and 36 females. All subjects were randomly assigned to 3 groups with an equal ratio of males and females in each group (26 males and 12 females/group). All participants signed a written informed consent form before starting the experiment. During the experiment, a small number of subjects in each group withdrew from the experiment due to changes in their physiological conditions, such as being diagnosed with COVID 19 or being temporarily unable to cooperate with the experimental schedule. The experimental procedure is described in Figure 1.

### 2.3. Experimental Design

The trial used a double-blind design, and subjects were randomly assigned to a placebo group (MCC, 2 capsules/day), a *L. paracasei* PS23 live bacteria group (1 × 10^10^ CFU/capsule, 2 capsules/day) (L-PS23), and a PS23 heat-killed bacteria group (1 × 10^10^ cells/capsule, 2 capsules/day) (HK-PS23), and the gender ratio was the same in each group. All participants were asked to supplement for 6 consecutive weeks and continued until 48 h after EIMD. During the experiment, participants visited the laboratory a total of 5 times. Body composition, basal blood biochemical parameters, and exercise performance were measured before placebo, L-PS23 and HK-PS23 supplementation, and six weeks after the intervention, followed by immediate EIMD. Biomarkers of muscle damage and indicators of inflammatory and oxidative damage were measured 3, 24, and 48 h after EIMD [32]. All participants were asked to maintain their daily diet throughout the study period and to record their dietary intake for a professional nutritionist to analyze. The basic information of the physical parameters of the subjects before the intervention is shown in Table 1.

### 2.4. Body Composition

All subjects were asked to fast for at least 8 h prior to testing. We used an InBody 570 device (In-body, Seoul, South Korea); the subject stood on the bottom electrode, held the sensing handle with both hands, did not speak or move during the measurement, and kept their arms open so that their torso was at a 30° angle. Body composition was measured using a multi-frequency principle within 60 s using a bioelectrical impedance analyzer (BIA) at 1, 5, 50, 260, 500 and 1000 kHz, as previously described [33].

### 2.5. Exercise-Induced Muscle Damage (EIMD)

We refer to the way in which EIMD was successfully induced in the past [32,34]. All subjects were asked to perform 100 maximal vertical jumps. This method involved jumping every 4 s, 10 times, as a group, for a total of 10 groups. All subjects were asked to bend their knees 90° during each squat, and each subject’s maximum jump height was marked as the goal. The researchers encouraged the subjects to try their best to reach the maximum height target with each jump.

### 2.6. Countermovement Jump Assessment (CMJ)

The CMJ test is one way to measure the maximum speed, strength, and explosiveness of the lower body. For this test, participants were asked to place their hands on their hips and stand with their feet on a Kistler force-measuring platform (9260AA, Kistler GmbH, Winterthur, Switzerland). Afterwards, they were instructed to squat until their knees were bent 90 degrees, then immediately jump as high as possible with maximum force. Each participant performed 3 replicates; we obtained CMJ data at the designated points and calibrated the instrument for each individual’s weight [34]. The measured parameters were rate of force development (RFD), relative peak force, and jump height.

### 2.7. Isometric Mid-Thigh Pull (IMTP)

IMTP is a reliable and efficient way to measure maximum force. A custom-made IMTP test kit (IMTP Rack, Kairos Strength, USA) and force plate (Model 9260AA, Kistler, Winterthur, Switzerland) were used. All subjects stood with their feet equal-width apart, with the bar between the thighs, the torso upright, the spine neutral, and the knees and hips at a 140° angle, pulling the bar with maximum force for 3–5 s. Before the test, subjects had to be familiar with the IMPT test method and exercises to ensure correct force and stable weight frequency [35]. Measurements were repeated at 2 min intervals for each test to reduce detection errors related to postural changes. The measured parameters were relative peak force and RFD.

### 2.8. Wingate Anaerobic Test (WAnT)

The subjects completed a full sprint in 30 s on an anaerobic power bike (894E, Monark Exercise AB, Vansbro, Sweden). The seat height could be adjusted for each participant, and toe clips prevented the feet from slipping off the pedals. We started measuring after warming up. When the subject sprinted at 120 rpm, a weight set to 7.5% of their body weight (for example, a 60 kg person would add 4.5 kg of resistance) automatically landed on the bike’s friction belt, creating additional resistance [34]. We continued asking the subject to sprint at full strength for 30 s. The measured parameters were relative mean power (W/kg), relative peak power (W/kg), and fatigue index (%).

### 2.9. Muscle Damage, Inflammation and Oxidative Damage Biomarker

Venous blood was collected from subjects at pre-EIMD, and 3, 24, and 48 h post-EIMD, for biomarker assessments, including muscle damage, inflammation and oxidative damage biomarkers. The CK and high-sensitivity CRP (hs-CRP) levels were analyzed by an autoanalyzer (AU 5820, Beckman Coulter Inc., CA, USA). The myoglobin and testosterone levels were analyzed by an autoanalyzer (DXI 800, Beckman Coulter Inc., CA, USA). The thiobarbituric acid reactive substances (TBARS) level was assessed with the TBARS Assay Kit (No. 10009055, Cayman Chemical Company, Ann Arbor, MI, USA).

### 2.10. Serum Biochemical Analysis

We collected blood from arm venous catheters before and after the 6-week sample intervention, analyzed liver function, renal function, blood lipids and glucose, and evaluated the subjects’ metabolic and health status to determine whether they were affected. All the subjects were asked to fast for at least 8 h and their blood serum was assessed with an autoanalyzer (Hitachi 717, Tokyo, Japan) for aspartate transaminase (AST), aminotransferase (ALT), total cholesterol (TC), triglyceride (TG), high-density lipid cholesterol (HDL-C), low-density lipid cholesterol (LDL-C), blood urea nitrogen (BUN), creatine (CREA), uric acid (UA), and glucose levels.

### 2.11. Statistical Analysis

All the data are expressed as mean ± SD. Statistical analyses were performed by SAS 9.0 (SAS Inst., Cary, NC, USA). Multi-group comparisons were analyzed by one-way analysis of variance (ANOVA). We used IBM SPSS Statistics version 24.1 (IBM Co., Armonk, NY, USA), and repeated measures ANOVA was used to compare placebo, L- PS23 and HK-PS23 at specified time points during recovery. Paired *t*-tests with Bonferroni adjustment were used to compare treatment differences with pre-EIMD at each time point. Statistical significance was set at *p* < 0.05.

## 3. Results

### 3.1. Effects on Dietary Intake and Body Composition before and after the 6-Week L-PS23 and HK-PS23 Intervention

Table 2 shows the subjects’ daily dietary intake (carbohydrate, protein, and fat) and total calories before the test and six weeks after the intervention. There was no significant difference between the groups, and there was no significant change in the before–after comparison within the group.

In terms of body composition, there were no significant differences in body weight, BMI, lean body mass (LBM) or fat body mass (FBM) between the groups before and after the six weeks of the intervention. However, in the placebo group after six weeks of intervention, compared with before, body weight (*p* = 0.0084), BMI (*p* = 0.0087) and FBM (*p* = 0.0044) significantly increased, but LBM decreased (*p* = 0.0072). In the HK-PS23 group after six weeks of the intervention, body weight (*p* = 0.0036) and BMI (*p* = 0.0028) decreased compared to before intervention. In addition, the degree of change in body composition was explored by calculating the difference before and after the intervention. The HK-PS23 group showed a significantly reduced body weight and BMI compared to both the placebo and L-PS23 groups (*p* < 0.05), and a significantly reduced FBM compared to the placebo group (*p* = 0.0109) (Table 3).

### 3.2. Effects of Biochemical Characteristics of Subjects before and after 6-Week L-PS23 and HK-PS23 Intervention

Before and after the intervention, all subjects underwent basic blood parameter analysis to understand the healthy physiological state of the subjects before the intervention, and to determine whether there were any adverse effects or side effects after the 6-week PS23 intervention. As shown in Table 4, we found that the liver function indexes (AST, ALT), renal function indexes (BUN, CREA, UA), blood lipid metabolism indexes (TC, TG, HDL-C, LDL-C) and blood sugar were all within the normal range, and there was no significant difference among the groups before or after the intervention. In addition, there were no significant changes in each group after the intervention compared with before the intervention. There were no adverse events related to intervention during the study.

### 3.3. Effect of L-PS23 and HK-PS23 Supplementation on Vertical Jump Height of Exercise Performance

**Figure 2:**
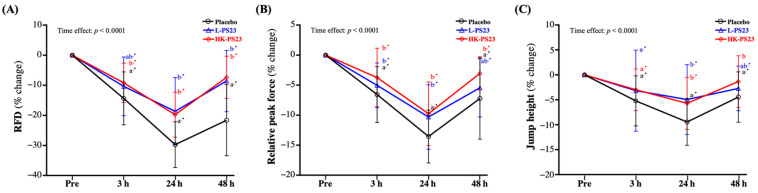
Effects of 6 weeks of L-PS23 and HK-PS23 supplementation on percentage change in CMJ test (**A**) RFD, (**B**) relative peak force, and (**C**) jump height. Data are expressed as mean ± SD for *n* = 35 subjects per group. Different superscript letters (a, b) indicate significant difference between groups at the same time point (*p* < 0.05). * indicates that each group is significantly different from pre-EIMD at different time points (*p* < 0.05). RFD, rate of force development.

At 3, 24, and 48 h post-EIMD, the percentage changes in RFD and relative force peak were significantly lower in the placebo, L-PS23, and HK-PS23 groups compared with pre-EIMD (*p* < 0.05). However, from the between-group comparisons, the L-PS23 and HK-PS23 supplementation groups showed significantly less RFD loss and faster recovery than the placebo group at 24 and 48 h after EIMD (*p* < 0.0001) (Figure 2A). As shown in Figure 2B, the L-PS23 and HK-PS23 supplementation groups showed significantly less relative force peak loss than the placebo group at 3 and 24 h post-EIMD (*p* < 0.05); however, only the HK-PS23 group showed significantly better recovery than the placebo group at 48 h post-EIMD (*p* = 0.0008). The jump heights in the L-PS23 and HK-PS23 supplementation groups showed significantly less loss than the placebo group at 24 h post-EIMD (*p* < 0.05), but only the HK-PS23 group showed significantly better recovery than the placebo group at 48 h post-EIMD (*p* = 0.0097) and showed no significant difference from pre-EIMD (Figure 2C). Significant time effects for RFD, relative peak force, and jump height were shown (*p* < 0.0001).

### 3.4. Effect of L-PS23 and HK-PS23 Supplementation on Isotonic Muscle Strength of Exercise Performance

**Figure 3:**
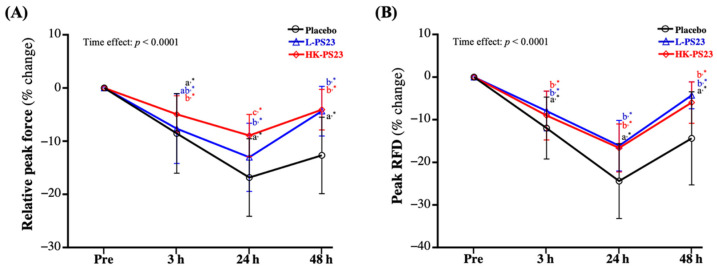
Effects of 6 weeks of L-PS23 and HK-PS23 supplementation on percentage change in IMTP test (**A**) relative peak force and (**B**) peak RFD. Data are expressed as mean ± SD for *n* = 35 subjects per group. Different superscript letters (a, b, c) indicate significant difference between groups at the same time point (*p* < 0.05). * indicates that each group is significantly different from pre-EIMD at different time points (*p* < 0.05). RFD, rate of force development.

At 3, 24, and 48 h post-EIMD, the percentage changes in relative force peak and peak RFD were significantly lower in the placebo, L-PS23, and HK-PS23 groups compared with pre-EIMD (*p* < 0.0001). However, the L-PS23 group showed a significantly lower loss of relative force peak at 24 and 48 h post-EIMD, and the HK-PS23 group showed significantly less loss of relative force peak at 3, 24, and 48 h post-EIMD, compared with the placebo group (*p* < 0.05) (Figure 3A). On the other hand, the L-PS23 and HK-PS23 groups showed significantly less loss of peak RFD at 3 and 24 h post-EIMD and showed significantly better recovery at 48 h post-EIMD than the placebo group (*p* < 0.05) (Figure 3B). Significant time effects for relative peak force and peak RFD were shown (*p* < 0.0001).

### 3.5. Effect of L-PS23 and HK-PS23 Supplementation on Anaerobic Exercise Performance

**Figure 4:**
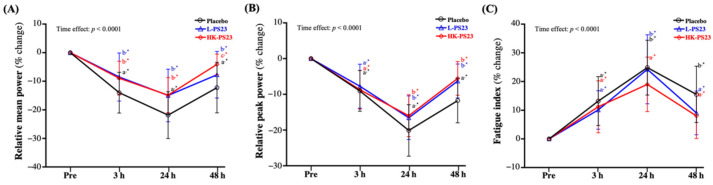
Effect of 6 weeks of L-PS23 and HK-PS23 supplementation on percentage change in Wingate test (**A**) relative mean power, (**B**) relative peak power, and (**C**) fatigue index. Data are expressed as mean ± SD for *n* = 35 subjects per group. Different superscript letters (a, b, c) indicate a significant difference between groups at the same time point (*p* < 0.05). * indicates that each group is significantly different from pre-EIMD at different time points (*p* < 0.05).

At 3, 24, and 48 h post-EIMD, the percentage changes in relative mean power and relative peak power were significantly lower, and fatigue index was significantly greater in the placebo, L-PS23, and HK-PS23 groups compared with pre-EIMD (*p* < 0.0001). The L-PS23 and HK-PS23 groups showed significantly less loss of relative mean power compared to the placebo group at 3, 24, and 48 h post-EIMD (*p* < 0.05) (Figure 4A). As shown in Figure 4B, the L-PS23 and HK-PS23 groups showed significantly less loss of relative peak power than the placebo group at 24 and 48 h post-EIMD (*p* < 0.05). On the fatigue index, only the HK-PS23 group showed a significantly lower increase at 24 h post-EIMD than the placebo group (*p* < 0.05), but at 48 h post-EIMD, the L-PS23 and HK-PS23 groups showed a significantly smaller increase than the placebo group (*p* < 0.05) (Figure 4C). Significant time effects for relative mean power, relative peak power, and fatigue index wereshown (*p* < 0.0001).

### 3.6. Effect of L-PS23 and HK-PS23 Supplementation on Muscle Damage and Inflammation Biomarkers

**Figure 5:**
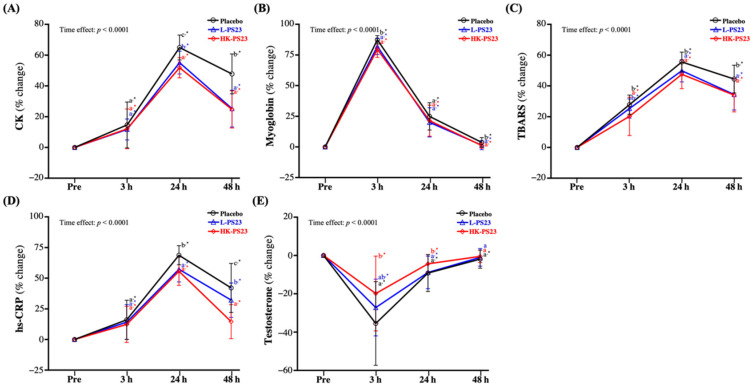
Effects of 6 weeks of L-PS23 and HK-PS23 supplementation on blood (**A**) CK, (**B**) myoglobin, (**C**) TBARS, (**D**) hs-CRP, and (**E**) testosterone. Data are expressed as mean ± SD for *n* = 35 subjects per group. Different superscript letters (a, b, c) indicate significant difference between groups at same time point (*p* < 0.05). * indicates that each group is significantly different from pre-EIMD at different time points (*p* < 0.05). CK, creatine kinase; TBARS, thiobarbituric acid reactive substances; hs-CRP, high sensitivity C-reactive protein.

In the current study, all groups showed significantly increased CK, myoglobin, TBARS, and hs-CRP at 3, 24, and 48 h post-EIMD (*p* < 0.05). The increases in CK activity at 24 and 48 h post-EIMD were significantly reduced in the L-PS23 and HK-PS23 groups compared to the placebo group (*p* < 0.05) (Figure 5A). The myoglobin increase ratio in the L-PS23 and HK-PS23 groups at 3 and 48 h post-EIMD were significantly lower than those in the placebo group (*p* < 0.05) (Figure 5B). As shown in Figure 5C, the TBARS increase ratio in the L-PS23 and HK-PS23 groups at 24 and 48 h post-EIMD were significantly lower than those in the placebo group (*p* < 0.05), but at 3 h post-EIMD, HK-PS23 was also significantly lower than in the placebo and L-PS23 groups (*p* < 0.05). The hs-CRP increase ratios in the L-PS23 and HK-PS23 groups at 24 and 48 h post-EIMD were significantly lower than that in the placebo group (*p* < 0.05); in particular, at 48 h post-EIMD, the HK-PS23 group was significantly lower than the L-PS23 group (*p* < 0.0001) (Figure 5D). As shown in Figure 5E, at 3 and 24 h post-EIMD, the percentage changes in testosterone were significantly lower in the placebo, L-PS23, and HK-PS23 groups compared with pre-EIMD (*p* < 0.0001). However, at 48 h post-EIMD, only the placebo group still yielded lower results than pre-EIMD, and the L-PS23 and HK-PS23 groups showed no significant difference from pre-EIMD. In addition, the HK-PS23 group at 3 and 24 h post-EIMD yielded significantly higher values than the placebo group (*p* < 0.05), and also significantly greater than those of the L-PS23 group at 24 h post-EIMD (*p* < 0.05).

## 4. Discussion

In recent years, the use of probiotics as a sports nutrition supplement has been gradually developed and become more highly valued. In addition to improving performance, it has been proven to improve the gastrointestinal tract and inflammation in athletes [16]. However, there have been no studies of *L. paracasei*-related strains on improving recovery from muscle damage. In the current study, we evaluated subjects who were without recent sport or resistance training experience and found that 6 weeks of administration of *L. paracasei* PS23, either live or heat-killed, reduced muscle strength loss, and blood biomarkers of muscle damage and inflammation, and accelerated recovery. Especially with HK-PS23, there were more significant benefits.

DOMS, a common symptom of EIMD, occurs when muscle fibers are damaged after strenuous exercise, and it persists for days after the muscle-damaging exercise [36]. It is usually characterized by decreased muscle strength and range of motion, localized inflammation, and muscle soreness, with increased levels of intramuscular proteins such as CK and myoglobin [37]. Despite a marked increase in EIMD markers after exercise, the long-term maintenance of high values may indicate a state of chronic fatigue and inadequate adaptation to training [38]. When fatigue occurs, it reduces muscle strength performance. Therefore, in strength and conditioning studies, vertical jumping has been used to evaluate athletes’ speed, maximal strength, explosive power, and anaerobic performance [39], and as one of the fatigue indicators of muscle damage and decreased muscle strength. [8]. Past research has shown that after strenuous exercise, the jumping ability decreased after 24 h, and then gradually recovered [40]. However, in functional and performance testing, maximal RFD and relative peak force are often used as more important indicators of muscle strength and power than maximal jump height [41]. Therefore, in our study, taking these three indicators as the main evaluation items, it was found that both L-PS23 and HK-PS23 could significantly prevent the decrease in jump-related strength performance after EIMD, and induced better recovery effects (Figure 2A–C). Past research has shown that giving soldiers two weeks of inactivated *Bacillus coagulans* combined with a soldier’s self-defense course can increase vertical jumping strength [42]. Another study on *B. coagulans* supplemented with 20 g of casein for two weeks also showed a trend of increased vertical jumping strength compared to the group supplemented with 20 g of casein alone. In addition, the anaerobic power performance, assessed via the Wingate test, was significantly improved [43]. In our previous study, we found that 4 weeks of supplementation with *Lactobacillus plantarum* PS128 could not only reduce muscle damage, inflammation, and oxidative stress after strenuous exercise, but also improved anaerobic power performance in triathletes [23]. According to the report, strength training can improve peak and average power and explosive power at maximum speed output in a 30-s Wingate test [44]. Therefore, many previous studies have also used the Wingate anaerobic test to evaluate the effects on lower-limb muscle strength and anaerobic strength after EIMD [42,43,45]. During a 30-s Wingate sprint, approximately 75% of the energy demand is provided by the anaerobic metabolism, through energy such as adenosine triphosphate (ATP) and creatine phosphate (PCr), resulting in maximum peak power output in a short period of time [46]. In a previous study on aging mice, PS23 was found to reduce aging-related mitochondrial alternation by regulating PGC1α, SIRT1, NRF1, and TFAM gene expression [27]. Past studies have shown that increased expression of the SIRT1 and PGC-1α signaling pathways can promote mitochondrial function and increase ATP [47]. Among them, transgenic increases in PGC-1α expression significantly increase glycogen synthesis and storage, and inhibit glycogenolysis [48], thereby promoting recovery and repair [49]. CMJ and Wingate results have also been shown to be reliably correlated. Therefore, we infer that six consecutive weeks of PS23 supplementation might improve the performance of Wingate’s anaerobic power (Figure 4A–C) through the above-mentioned related mechanisms, but further confirmation is needed. The IMTP test has been shown to be a valid and reliable test of maximal lower extremity strength, which is highly correlated with exercise performance, and is used for changes in explosive power and as a marker of fatigue [50]. Among them, RFD is defined as the ability to generate force at a given time during rapid voluntary contractions, while it is often used to assess blast strength [51]. A past study has noted a significant drop in strength, 24 h after exercise, returning to baseline values at between 48 and 72 h [52]. However, three consecutive weeks of supplementation with the probiotics *Streptococcus thermophilus* FP4 and *Bifidobacterium breve* BR03 reduced the degree of decline in exercise performance and range of motion following muscle-damaging exercise [53]. This may be related to the ability of probiotics to increase amino acid absorption [54]. Acute exercise combined with protein or amino acid intake can enhance muscle protein anabolic response by activating the mTORC1 pathway, which is beneficial to promoting post-exercise recovery [55]. In this study, although the supplementation with L-PS23 or HK-PS23 probiotics alone may promote the absorption and utilization of protein in the diet, thereby promoting muscle recovery (Figure 3A,B), further research is still needed.

Blood-related indicators are also one of the most effective indirect areas of focus for assessing muscle damage and inflammation. CK is typically elevated after exercise and is frequently used as a biomarker of skeletal muscle tissue damage [56]. After muscle damage, the CK concentration begins to increase within 2–12 h, peaks within 24–72 h, and gradually decreases to recovery [57]. In a recent study, elevated CK was shown to be associated with fatigue, and was strongly associated with the decrease in relative work output observed during eccentric exercise of the muscle-damaging rectus femoris in consecutive groups [58]. Another commonly used indicator of muscle damage is myoglobin. During severe muscle damage, elevated levels of myoglobin are released into the bloodstream, which is often associated with the release of an enzyme, and this can rise rapidly within hours of injury. It is the earliest marker of myocardial infarction and rhabdomyolysis [59]. Currently, there are no complete studies investigating the mechanism by which probiotic supplementation improves CK and myoglobin. However, in a retrospective study, probiotics were found to help increase short-chain fatty acids (SCFA) in the gut, which in turn increased glycogen concentration and protein digestibility, and promoted muscle repair and recovery [60]. A past animal study also showed that *L. plantarum* Tana supplementation for four weeks significantly improved exercise performance and reduced post-exercise blood lactate and blood ammonia levels, as well as CK activity [61]. In the results of this study, supplementation with L-PS23 or HK-PS23 not only slowed post-exercise CK or myoglobin elevation, but also helped to accelerate recovery (Figure 5A,B). In addition to muscle tissue damage, strenuous exercise may significantly increase oxygen consumption while generating ROS [62]. ROS react with polyunsaturated fatty acid side chains of biological membranes, and macromolecular substances such as nucleic-acid-related phospholipids, enzymes, and membrane receptors to form lipid peroxidation products, thereby changing the fluidity and permeability of cell membranes, resulting in changes in cell structure [63]. At this point, free radicals are produced, and cytokines are excreted, leading to oxidative damage [64]. On the other hand, when the muscles contract violently, neutrophil infiltration can also be induced, leading to acute muscle inflammation, possibly leading to leukocyte infiltration and elevated levels of inflammatory cytokines [64,65]. Previous studies have pointed out that the antioxidant mechanisms of lactic acid bacteria include scavenging oxidant compounds, reducing activity, chelating metal ions, preventing intestinal ROS formation, and regulating the expression of nuclear factor erythrocyte 2-related factor 2 (Nrf 2); through the promotion of SCFA they can reduce circulating endotoxins, inflammation, and oxidative stress [66,67,68]. In a systematic review and meta-analysis, probiotic supplementation significantly increased total antioxidant capacity (TAC) and decreased the oxidation marker MDA [69]. In a study of cyclists, six weeks of supplementation with mixed strains (*Bifidobacterium longum* CECT 7347, *Lactobacillus casei* CECT 9104, and *Lactobacillus rhamnosus* CECT 8361) was found to significantly reduce post-exercise MDA production compared to placebo [70]. This is similar to our findings (Figure 5C). The acute-phase response evoked after strenuous exercise results in cytokines being secreted by many tissues, such as IL-6, increasing the production of CRP in the liver and its release into the blood. Therefore, there will be short-term, transient elevations in serum CRP [71]. However, exercise training can reduce cytokine production by adipose tissue, endothelial cells, and blood monocytes, and improve endothelial function, insulin sensitivity and antioxidant effects, thereby reducing CRP levels [72]. In a previous study, probiotic intake significantly decreased hs-CRP and MDA, and increased GSH and TAC, in patients with diabetic nephropathy, with beneficial effects on biomarkers of inflammation and oxidative stress [73]. In our past research, it was also found that supplementation with *L. plantarum* PS128 in triathletes reduced post-exercise CK and pro-inflammatory cytokines and reduced oxidative stress [43]. This suggests that the L-PS23 and HK-PS23 supplementation for 6 weeks in this study can protect muscle damage and reduce oxidative stress and hs-CRP after EIMD (Figure 5A–D). Anabolic and catabolic hormone statuses change dramatically after exercise [74]. Among them, testosterone is an androgenic and anabolic hormone secreted by the hypothalamic–pituitary–testis axis, and elevated levels indicate an anabolic state, whereas the opposite indicates impaired exercise performance leading to fatigue [75]. During exercise, testosterone is a recognized androgen that maintains anabolism by promoting bone development and protein synthesis within the musculoskeletal system; thus, testosterone anabolism affects skeletal muscle mass and function [76]. However, past research has shown that after two weeks of high-intensity wrestling training, which causes severe muscle damage, wrestlers have 30 percent lower blood testosterone levels and increased CK activity, which weakens and delays skeletal muscle regeneration [77]. This may be because testosterone enters the skeletal muscle during exercise and binds to androgen receptors, providing increased anabolic stimulation to promote intramuscular skeletal development and protein synthesis [76]. In past animal experiments, supplementation with *L. plantarum* TW1-1 was observed to significantly improve diethylhexylphthalate-induced serum testosterone concentrations in mice [78]. Although no effect of probiotic supplementation on post-training testosterone in humans has been observed in previous studies, there may be a difference in training level or EIMD [41]. However, in the present study, testosterone concentrations were significantly reduced in all groups 3 h after EIMD; nonetheless, supplementation with L-PS23 and HK-PS23 led to significantly reduced loss and faster recovery benefits, especially in the HK-PS23 group (Figure 5E).

In our study, we found that both L-PS23 and HK-PS23 supplementation can improve muscle damage, inflammation, etc., after EIMD, as well as accelerating muscle damage repair, strength, and fatigue recovery, and HK-PS23 supplementation is better than L-PS23. The health benefits of probiotics are strain- and dosage-dependent; however, due to storage method, time, etc., live bacteria may be gradually damaged or killed, which reduces the total number of live bacteria [79]. In contrast, inactivated probiotics have the advantages of being safer, having a longer shelf-life, being easier to transport and store, and they are the focus of the current research trend in anti-inflammatory and immune-boosting supplements [80]. Heat-killed probiotics contain inactive bacterial cells and/or metabolites produced by live probiotics, such as exopolysaccharides, peptidoglycan, lipoteichoic acid, SCFA, and amino acids [81]. A previous study showed that non-viable bacteria and bacterial fractions could pass through the mucus and stimulate epithelial cells more efficiently than live bacteria [82]. Compared with live bacteria, heat-killed *L. plantarum* significantly inhibited brain injury and neuroinflammation (reduced IL-1β and IL-6, increased IL-4, and IL-10, and prevented *Salmonella enterica* Typhimurium infection-induced reduction in SCFA) [19]. Furthermore, a previous study showed that augmentation of the AMPK-PGC-1α signaling pathway by *Bifidobacterium breve* B-3HK supplementation enhanced mitochondrial biosynthesis and activated Akt-mTOR protein synthesis signaling, resulting in increased muscle mass [83]. In one trial of athletes, heat-killed *Lactococcus lactis* JCM 5805 supplementation for 14 days followed by high-intensity exercise resulted in significant improvements in respiratory and fatigue symptoms, although there were no significant differences in markers of muscle damage and stress [22]. These may be the reasons why HK-PS23 was superior to the L-PS23 group in muscle damage and strength recovery in this trial, although L-PS23 also showed significant benefits. However, further studies and comparisons are needed to explore the possible mechanistic differences.

Although many studies have shown the feasibility of using probiotics as a sports nutrition supplement, most of the research is still focused on improving immunity, the respiratory tract, or improving sports performance in athletes. There has been little research as yet on the recovery benefits for muscle damage. The results of this study confirm the protective, recovery, and improvement benefits of PS23 supplementation after muscle damage. However, we still recommend that appropriate supplementation with *L. paracasei* PS23 may help improve muscle strength and recovery from damage after overtraining or high-intensity competition. Despite this, this study still has some limitations, including the following: (1) The subjects of this study were all non-athletic students from the same university. Although their living conditions are similar, they may not be fully representative of all aspects. (2) This study mainly explored the differences between different samples in improving muscle damage and strength recovery, so male and female subjects were included in the same group for comparison. There are no particular restrictions on the recruitment of male and female subjects, considering gender equality. However, male subjects participated more actively, so the ratio of males to females in this trial was 7:3, and they were evenly distributed among the groups. Although we know that there are differences in strength or physiology between male and female subjects, the benefits of treatments between different samples can be observed across groups. More subjects can be included in the future to further compare the effects and mechanisms of this trial sample in both male and female subjects. (3) Since the blood sample collection and physical fitness tests in this trial may place a greater burden on the subjects, Therefore, more tests are needed in the future, such as looking at other biochemical parameters of aerobic exercise or exercise fatigue. (4) To further understand the cellular mechanisms behind our findings, muscle biopsies or in vitro animal studies may be required. Based on the above, we need a broader perspective to confirm our findings and we expect to further explore the mechanisms of cell injury and recovery, and the role of the muscle-gut axis by analyzing the gut microbiota.

## 5. Conclusions

In conclusion, we showed that six consecutive weeks of L-PS23 or HK-PS23 supplementation significantly reduced muscle force loss, blood biomarkers of muscle injury, and inflammation after muscle damage, while being beneficial in accelerating recovery. Therefore, we believe that probiotics have potential benefits not only in improving exercise performance, but also in improving protection and recovery from exercise-induced muscle damage. Further comparison of the recovery effect of *L. paracasei* PS23 on muscle injury in men and women is required in the future. Further focused research is also required, such as in vitro or animal experiments and gut bacteria analysis, to explore the way to aid muscle cell activity or regeneration, to find out the mechanism of *L. paracasei* PS23 in promoting muscle injury recovery.

## Figures and Tables

**Table 1:** Participant characteristics and physical fitness test at baseline.

Basic Information	Parameter (Unit)	Placebo	L-PS23	HK-PS23
Physical characteristics	Age (years)	21.6 ± 2.0	21.4 ± 1.3	21.8 ± 2.5
	Height (cm)	172 ± 9	172 ± 9	171 ± 9
	Weight (kg)	69.4 ± 12.4	69.7± 11.0	66.4 ± 11.6
	BMI (kg/m^2^)	23.2 ± 2.8	23.5 ± 2.4	22.7 ± 2.6
CMJ	RFD (N/kg/Sec)	8.11± 0.95	8.03 ± 1.03	7.96 ± 1.08
	Relative force peak (N/kg)	15.11 ± 2.12	15.23 ± 2.22	15.09 ± 2.55
	Jump height (cm)	30.7 ± 6.0	31.6± 7.6	32.0 ± 6.5
IMTP	Relative Peak Force (N/kg)	15.57± 2.32	15.31 ± 3.06	15.46 ± 2.51
	pRFD (N/Sec)	9244 ± 1777	9219 ± 1821	9102 ± 1520
Wingate	Relative mean power (W/kg)	6.17± 0.91	6.53 ± 0.94	6.45 ± 0.97
	Relative peak power (W/kg)	9.61 ± 1.59	9.40 ± 1.76	9.38 ± 1.73
	Fatigue index (%)	48.6 ± 3.9	47.8 ±4.2	48.1 ± 4.6

Data are presented as mean ± SD. BMI, body mass index. CMJ, countermovement jump; IMTP, isometric mid-thigh pulled; RFD, rate of force development.

**Table 2:** Subject’s dietary intake before and after the 6-week L-PS23 and HK-PS23 intervention.

Dietary Intake	Before			After		
	Placebo	L-PS23	HK-PS23	Placebo	L-PS23	HK-PS23
Carbohydrate (g/day)	214 ± 69	213 ± 37	208 ± 34	214 ± 70	215 ± 48	213 ± 36
Protein (g/day)	152 ± 23	155 ± 20	147 ± 27	149 ± 23	155 ± 19	149 ± 23
Fat (g/day)	92 ± 5	93 ± 6	90 ± 5	93 ± 5	93± 9	92 ± 6
Total calorie (kcal/day)	2287± 313	2304 ± 163	2232 ± 153	2289 ± 315	2319 ± 219	2274 ± 194

Data are presented as mean ± SD.

**Table 3:** Subject’s body composition before and after the 6-week L-PS23 and HK-PS23 intervention.

Parameters	Before			After			Delta		
	Placebo	L-PS23	HK-PS23	Placebo	L-PS23	HK-PS23	Placebo	L-PS23	HK-PS23
BW (kg)	69.4 ± 12.4 ^a^	69.7 ± 11.0 ^a^	66.4 ± 11.6 ^a^	70.1 ± 12.9 ^a,^*	70.0 ± 11.0 ^a^	65.6 ± 11.7 ^a,^*	0.7 ± 1.4 ^b^	0.3± 2.3 ^b^	−0.8 ± 1.6 ^a^
BMI (kg/m^2^)	23.2 ± 2.8 ^a^	23.5 ± 2.4 ^a^	22.7 ± 2.6 ^a^	23.5 ± 3.0 ^a,^*	23.6 ± 2.5 ^a^	22.4 ± 2.7 ^a,^*	0.2 ± 0.5 ^b^	0.1 ± 0.8 ^b^	−0.3 ± 0.5 ^a^
LBM (kg)	31.8 ± 6.7 ^a^	31.7 ± 6.3 ^a^	30.2 ± 6.2 ^a^	31.5 ± 6.7 ^a,^*	31.7 ± 6.4 ^a^	30.2 ± 6.0 ^a^	−0.3 ± 0.6 ^a^	0.0 ± 0.9 ^a^	−0.1 ± 0.6 ^a^
FBM (%)	20.0 ± 7.0 ^a^	20.1 ± 7.9 ^a^	19.3 ± 6.5 ^a^	20.8 ± 6.7 ^a,^*	20.2 ± 8.5 ^a^	19.1 ± 6.0 ^a^	0.8± 1.5 ^b^	0.1 ± 1.4 ^ab^	−0.2 ± 1.7 ^a^

Data are presented as mean ± SD. Different superscript letters (a, b) indicate significant difference among groups at the same time point, *p* < 0.05. * Indicates a significant effect after intervention compared to before intervention, *p* < 0.05. Delta is the value after intervention minus before intervention. BW, body weight; BMI, body mass index; LBM, lean body mass; FBM, fat body mass.

**Table 4:** Subject’s blood biochemical parameters before and after the 6-week L-PS23 and HK-PS23 intervention.

Parameters	Before			After		
	Placebo	L-PS23	HK-PS23	Placebo	L-PS23	HK-PS23
AST (U/L)	21 ± 6	22 ± 7	21 ± 7	21 ± 6	22 ± 6	121 ± 6
ALT (U/L)	20 ± 6	22 ± 7	20 ± 7	20 ± 4	22 ± 7	21 ± 7
TC (mg/dL)	179 ± 16	173 ± 18	174 ± 22	174 ± 21	178 ± 15	172 ± 18
TG (mg/dL)	81 ± 24	84 ± 24	81 ± 18	82 ± 22	85 ± 22	82 ± 17
HDL-C (mg/dL)	57.5 ± 9.3	56.7 ± 8.5	57.5 ± 7.6	58.0 ± 9.5	56.8 ± 9.1	58.2 ± 8.3
LDL-C (mg/dL)	96.5 ± 17.7	98.2 ± 15.2	97.1 ± 17.3	96.5 ± 18.0	97.2 ± 15.2	96.0 ± 16.5
BUN (mg/dL)	13.8 ± 2.5	14.9 ± 2.7	14.7 ± 3.1	13.5 ± 2.9	14.5 ± 2.3	14.4 ± 2.5
CREA (mg/dL)	1.10 ± 0.10	1.10 ± 0.11	1.09 ± 0.12	1.06 ± 0.12	1.10 ± 0.11	1.07 ± 0.10
UA (mg/dL)	5.7 ± 0.9	5.6 ± 0.9	5.4 ± 1.0	5.5 ± 0.8	5.6 ± 1.0	5.3 ± 1.1
Glucose (mg/dL)	86 ± 6	84 ± 6	84 ± 8	84 ± 6	84 ± 7	84 ± 7

Data are presented as mean ± SD. AST, aspartate aminotransferase; ALT, alanine transaminase; TC, total cholesterol; TG, triacylglycerol; HDL-C, high-density lipid cholesterol; LDL-C, low-density lipid cholesterol; BUN, blood urea nitrogen; CREA, creatinine; UA, uric acid.

## Data Availability

The data presented in this study are available within the article.
